# Complementary feeding and food handling practices among caregivers in a semi-urban setting in Northern Uganda

**DOI:** 10.4314/ahs.v24i3.36

**Published:** 2024-09

**Authors:** Elizabeth Atim, Abel Atukwase, Margaret Kabahenda

**Affiliations:** Department of Food Technology and Nutrition, College of Agricultural and Environmental Sciences, Makerere University, Kampala, Uganda

**Keywords:** Complementary feeding practices, food handling, diet quality, food safety, children, Uganda

## Abstract

**Background:**

Complementary feeding practices influence the nutritional status and health of children, especially in developing countries where sub-optimal infant feeding practices are the major cause of childhood undernutrition, morbidity, and mortality. This study sought to characterize the adequacy of complimentary feeding and food handling practices among caregivers of children aged 6-23 months in a semi-urban setting.

**Methods:**

Exploratory cross-sectional study was conducted in Adyel division, Lira district in Northern Uganda among 127 child-caregiver pairs randomly selected from three villages. Caregivers were interviewed about their child-feeding practices, complementary foods used, and common practices in handling and preparation of foods used as complementary feeds.

**Results:**

Overall, children's feeding patterns met the recommended dietary intake when assessed using the child feeding index (CFI) whereby children aged 6-8, 9-11, and 12-23 months scored 8.03, 9.17, and 7.59, respectively. However, most children were complemented with foods from the household meals and dietary diversity was limited by frequent use of food leftover from the previous meals.

**Conclusions:**

Inappropriate meal patterns and food handling practices identified are likely to compromise child nutrition and health. Nutrition education is needed to improve the caregivers' child-feeding and food handling practices within their socio-economic and cultural context.

## Introduction

Strategies designed to enhance complementary feeding have been identified as critical to improving the nutritional status and health of children^1^, especially in developing countries where sub-optimal child-feeding practices influence childhood nutrition and growth^2^,^3^. To improve child feeding practices, the Uganda infant and young child feeding (IYCF) Guidelines Policy^4^ states that parents shall be counselled and supported to introduce adequate, safe, and appropriately feed complementary foods at 6 months of the infant's age while they continue breastfeeding for up to 2 years or beyond – which is in line with World Health Organisation guidelines^5^. However, it remains unclear whether children in Uganda are being fed as recommended.

The WHO core indicators of optimum nutrition for children above 6 months include: continued breastfeeding; the introduction of age-appropriate complementary foods; minimum dietary diversity; minimum meal frequency; minimum acceptable diet; and consumption of iron-rich or iron-fortified foods^2^. Among the key indicators, minimum dietary diversity is measured based on consumption of at least 4 or more varieties of foods from the 7 food groups in a 24 hour time period; Minimum meal frequency is measured in terms of receiving complementary foods the minimum recommended number of times in 24 hours (for breastfed children, the frequency should be at least 2 times for 6 to 8 months, and at least 3 times for children 9 to 23 months of age while for non-breastfed children, it should be at least 4 times)^6^. Hence, it is important to assess children's diets to determine the level at which these recommendations are met. Besides diet quality and feeding frequency, inappropriate food handling practices are a major risk of childhood malnutrition in developing countries as these practices predispose young children to recurrent infections^7^–^10^. In many rural contexts, children adhere to near optimal feeding patterns; however, children remain vulnerable to infections associated with unhygienic meal preparation practices such as handling food with unwashed hands, using unsafe water, or serving food using contaminated containers^11^–^13^.

Since the complementary feeding practices in the different regions of Uganda are not fully documented, and compliance with the WHO and national IYCF recommendations remains unclear, this study sought to characterize the adequacy of complimentary feeding and food handling practices among caregivers of children aged 6 to 23 months in a semi-urban setting in Northern Uganda.

## Methods

This was an exploratory cross-sectional survey conducted in Adyel division which is a semi-urban area in Lira district, Northern Uganda. The primary target population consisted of children of 6 to 23 months of age while their primary caregivers were the secondary targets for assessment of child-feeding practices. Hence, all children aged 6 to 23 months whose caregivers were willing to participate in the study were eligible for enrolment. Children with chronic health complications that require adherence to special feeding regimens were not included in the study. Since there was no available literature on determinants of complementary feeding in Uganda at the time of conceptualization of this study, the number of children included in the study was determined using the formula by Daniel^14^, whereby the prevalence of stunting (8.4%) was used to estimate chronic childhood undernutrition – which in turn is documented to be synergistically associated with episodes of diarrhoea among young children^7^. Hence the prevalence of inappropriate child-feeding and food handling practices (p) were also estimated at 8.4%. Based on a 5% desired level of absolute precision (d) and an estimated incomplete or missing data rate of 7%, a sample size of 127 child-caregiver pairs was needed for the study. Three villages within Adyel division were randomly selected and the study participants were obtained by simple random probability sampling, from a sampling frame that comprised of lists of households with children aged 6 to 23 months old in the three villages. The study protocol was reviewed and approved by the Uganda National Council of Science and Technology (Registration number HS1544). Informed consent was also obtained from all participants prior to data collection.

A structured, interviewer-administered, questionnaire was used to determine child-feeding practices, the commonest foods used as complementary feeds, and the common methods used in handling and preparation of complementary feeds. Since all the children enrolled in the study were 6 months and older, exclusive breastfeeding was assessed by asking caregivers to report how long they had exclusively breastfed the index child. Caregivers were also asked whether they fed their children using a bottle or not. The multiple pass 24-hour dietary recall was used to interview caregivers about children's dietary intake and this data were used to compute the mean minimum dietary diversity (MDD) and minimum meal frequency (MMF). The 24-hour recall data was also used to compute the child feeding index (CFI) scores for the age groups of 6-8, 9-11, and 12-23 months) as described by Ruel and Menon^15^.

A modification of the Delta NIRI Food Frequency Questionnaire^16^ composed of 71 foods used in the study area was used to interview caregivers on whether their children had consumed each food or not during the past 7 days. Individual foods were then grouped into food groups, namely: Staples, pulses, dairy products, flesh meats, eggs, vitamin A-rich fruits and vegetables, other fruits and vegetables, and oils. The sums of number of food groups consumed by each child were then used to compute the minimum dietary diversity (MDD) score. Additionally, individual foods listed on the food frequency questionnaire were also grouped into four CFI food groups (namely Staples, Meats, Eggs/fish/poultry, and Milk) and the scores were weighted to derive the food group frequency for the past 7 days indicator^15^.

The mean CFI for the different age groups (6-8, 9-11, and 12-23 months) were computed by summing up each age groups scores on breastfeeding, use of bottle, dietary diversity in the past 24 hours, meal frequency in the past 24 hours, and food frequency for the past 7 days. The total CFI score was then used to determine the proportion of children with adequate dietary intake and the mean diet adequacy corresponding to each stage of child development^15^.

Open-ended questions were included in the structured questionnaire and used to generate more in-depth qualitative data on caregivers' perceptions and practices related to child-feeding and food handling. The qualitative data collected on caregivers' beliefs, food handling and preparation practices, and their child-caregiving practices were summarized into common themes identified through literature review and the frequency of mention. The quantitative data was coded and analysed using the Statistical Package for Social Scientists (IBM SPSS version 21). Descriptive statistics such as means, frequencies and percentages were computed to characterize the participants' socio-demographic factors and engagement in specific child feeding and food handling practice.

## Results

### Social demographic characteristics of the study population

Table 1 shows the socio-demographic characteristics of the study population. A total of 127 child-caregiver pairs were engaged in this study. Mean age of children was 14.3 months (Min = 6 months; Max = 23 months); and the majority were females (56.7%). The mean age of caregivers was 24.9 years (Range: 14 -70 years) and most of the caregivers (92.1%) were the biological mothers of children who participated in the study.

**Table 1:** Socio-demographic characteristics of study participants

Characteristic	Variables	Villages (%)			Gender (%)		Overall % Study Population
							(N = 127)

		Kirombe North A (n=45)	Kirombe North B (n=39)	Starch Factory B (n=43)	Male (n=55)	Female (n=72)	
***Characteristics of caregivers:***							
Age (years)	Less than 20	15.6	25.6	11.6	18.2	16.7	17.3
	20 -24	40	41	37.2	45.5	34.7	39.4
	25 – 29	31.1	23.1	30.2	18.2	36.1	28.3
	30 – 34	8.9	2.6	7	9.1	4.2	6.3
	35 -39	2.2	2.6	11.6	5.5	5.6	5.5
	40 -44	0	2.6	0	1.8	0	0.8
	45 -49	0	2.6	2.3	0	2.8	1.6
	above 49	2.2	0	0	1.8	0	0.8
Sex	Male	2.2	2.6	2.3	-	-	2.4
	Female	97.8	97.4	97.7	-	-	97.6
Relationship to child engaged in study	Mother	91.1	92.3	93	92.7	91.7	92.1
	Father	0	2.6	2.3	**2** 0.0	2.8	1.6
	Grandmother	2.2	2.6	0	3.6	0	1.6
	Sibling	2.2	0	2.3	1.8	1.4	1.6
	Other relative	4.4	2.6	2.3	1.8	4.2	3.2
***Characteristics of children:***							
Age (months)	6 -8	26.7	20.5	20.9	25.5	20.8	22.8
	9 -11	8.9	5.1	14.0	14.5	5.6	9.4
	12 -23	64.4	74.4	65.1	60.0	73.6	67.7
Sex	Male	44.4	35.9	48.8	-	-	43.3
	Female	55.6	64.1	51.2	-	-	56.7

### Breastfeeding and complementary feeding practices

Almost three quarters (73.2%) of the caregivers were still breastfeeding their children at the time of this study and 60.6% reported that they had exclusively breastfed their children for the recommended first 6 months of life. Although 73.2% of the children were still breastfeeding, 47% of caregivers reported giving their children cow's milk in the day preceding the survey – which indicates that about a quarter of children were being fed both cow's milk and breastmilk.

Table 2 shows that 87.4% of the children had MDD of at least 3 food groups per day; however, children's diets were limited in animal source foods. The commonly consumed food groups were staples (bananas, cassava, and maize meal), pulses (mostly beans and groundnuts), sugars and oils.

**Table 2:** Children's Feeding Patterns across Child Age groups and by Sex

Feeding Practice	%Children by age group in months			%Children by sex		Overall study population

	6 to 8	9 to 11	12 to 23	Male	Female	
***Breastfeeding***						
Yes	96.6	100	61.6	76.4	70.8	73.2
Not breastfeeding	3.4	0.0	38.4	23.6	29.2	26.8
***Food groups consumed in 24 hours***						
1	3.4	0.0	0.0	0	1.4	0.8
2	24.1	25.0	5.8	9.1	13.9	11.8
3	51.7	50.0	19.8	38.2	23.6	29.9
≥4	20.6	25.0	74.3	52.7	61.1	57.5
***Number of meals consumed by child in 24 hours***						
1	13.8	25.0	3.5	7.3	8.3	7.9
2	27.6	16.7	9.3	20.0	9.7	14.2
3	41.4	50.0	54.7	49.1	52.8	51.2
≥4	17.2	8.3	32.6	23.6	29.2	26.7
Mean age when introduced to complementary feeds	5.5	5.3	5.0	5.6	5.8	5.7

Based on the World Health Organization meal frequency guidelines^6^, to achieve the minimum meal frequency (MMF), breastfeeding children of 6-8, 9-11, and 12-23 months should be fed at least 2, 3, and 3 meals per day, respectively; while children of the same age that were not breastfeeding should have an extra one meal (or be fed 3, 4 and 4 meals per day respectively). In this study, 13.8%, 41.7%, and 34.8% of children 6-8, 9-11, and 12-23 months had a fewer number of meals than recommended in the WHO IYCF guidelines^6^.

### Commonly used complementary foods

This study revealed that children in Adyel division were majorly fed on household meals, which is a widespread practice across the country. Only 13.4% of the respondents reported that they prepared separate meals for their young children while 86.6% of the children (6-23 months) engaged in this study were complemented with foods from the household meals. Caregivers' recalls of children's dietary intake indicate that almost all children (97%) regularly consumed oils used in preparing household meals and 83% were provided with sweetened beverages during the 7-day period preceding the survey. Foods in the Cereals and grains plus the Legumes, nuts and seeds comprise the major complementary feeds in Uganda and these were consumed by 66% and 61% of the children, respectively. Nutrient-dense foods from the meats, dairy products, fruits, and vegetables groups were consumed by only 45%, 47%, 42% and 53% of the children, respectively.

### Adequacy of children's diets

All the children in the study had been introduced to complementary foods and the mean age at which children were introduced to complementary feeding was approximately 6 months (Mean = 5.69 months). Almost two thirds of caregivers (60.6%) introduced complementary foods at 6 months while 33.1% introduced complementary foods before the children were six months old. Some of the reasons given for early introduction of complementary foods were: i) death or separation of the biological mother; ii) mother having illnesses such as HIV and breast infections; iii) the inability of the mother to go to work with the child; and iv) mothers' perceptions that their children will not accept other foods in future if the complementary feeds were not introduced early. Possible delays in initiation of complementary feeding were documented among 6.3% of the caregivers that reported introducing complementary foods when their children were between 7 to 9 months of age.

Overall, 83% of children assessed had the minimum recommended CFI score of 6 points and the mean CFI scores for all age groups were within the acceptable range of 6 to 12 (see Table 3). Children's diets were compromised by the types of food groups selected and the number of meals provided. Based on the CFI scores, children 6-8 months had an average of 2 meals/day, those 9-11 months had 1-2 meals/day, while those 12-23 months had 2-3 meals/day. This indicates inadequate meal frequency. Quantification of children's dietary intake using the CFI food groups (Table 3) revealed limited frequency in consumption of eggs, fish and poultry among all children. Children aged 6-8 months and 12-23 months also had low mean frequency of meat consumption of 1 to 3 times/week.

**Table 3:** Adequacy of complementary feeding practices based on child feeding index (CFI)

Variables	Mean CFI score by child age group [maximum possible score]		

	6-8 months 29)	(n = 9-11 months (n = 12)	12-23 months (n = 86)
Breastfeeding	1.97 [2]	2.0 [2]	1.0 [1]
Uses bottle	1.0 [1]	1.0 [1]	0.59 [1]
Dietary diversity (in past 24 hours)	1.0 [2]	1.0 [2]	2.0 [2]
Food group frequency in past 7 days:			
Milk	-	-	1 [2]
Egg/fish/poultry	0 [2]	1 [2]	1 [2]
Meats	1 [2]	2 [2]	1 [2]
Staples (grains or tubers)	1 [1]	1 [1]	-
Total for food group frequency	**2 [5]**	**4 [5]**	**3 [6]**
Meal frequency (past 24 hours)	2 [2]	1 [2]	1 [2]
**Mean total CFI scores**	**7.97 [12]**	**9.17 [12]**	**7.59 [12]**

Adapted from Ruel and Menon (2002]

Caregivers indicated that the foods given to children were the foods available locally and this was determined by the seasonal availability of different food items. During this study, maize and beans were in harvest and thus were the foods available to most households. Breastfeeding was the largest contributor to the CFI scores observed for all child age groups. The use of bottles is not recommended but was common among children 12 to 23 months.

### Complementary Food Handling Practices

In this study, 85% of caregivers prepared food for later use (commonly referred to as leftover food). However, the caregivers reported that ‘leftover food’ was often not appropriately warmed before serving it to children (Table 4). Information in Table 4 further reveals unsafe food handling practices such as caregivers not washing their own hands after cleaning children who had defecated (70%) and not washing children's hands before serving them food (84%) were common among the study respondents.

**Table 4:** Prevalence of unsafe food handling practices among caregivers

Risky practice	Proportion (%) of caregivers with child in specified age group that engage in practice			Total caregivers (%)

	6-8 months	9-11 months	12-23 months	
**Complementary food handling:**				
Kept cooked food for later meals	79.3	91.7	86.1	**85.0**
Did not warm food kept for later use	4.3	9.1	4.1	**4.6**
Inadequate warming of leftover food	75.9	83.3	82.6	**85.0**
**Did not wash own hands:**				
Before food preparation	37.9	66.7	41.9	**43.3**
Before serving food to children	17.2	16.7	17.4	**17.3**
After using the toilet	34.5	41.7	32.6	**33.9**
After cleaning child that had defecated	57.1	83.3	72.1	**69.8**
**Did not wash child hands:**				
Before eating	65.5	91.7	89.5	**84.3**
After defecating	24.1	8.3	36.0	**30.7**
When visibly dirty	34.5	16.7	41.9	**37.8**

The practice of preparing food for later use is expected to improve the adequacy of children's diets by increasing child-feeding frequency or attainment of the minimum meal frequency (MMF). This is especially important in settings where the caregivers (mostly women) often spend much of their time engaged in farming activities away from home and have limited time dedicated to cooking. Besides, the food items commonly were hardly processed which increases food preparation and cooking time and thus makes meal preparation a laborious task. Results in Table 5 indicate that 85% of respondents reported that they kept some cooked food for later meals (commonly called leftovers as a social norm, to save cooking fuel, and to avoid food wastage. The remaining15% were against the practice of feeding children on leftover food because they preferred to prepare food that was just enough for a particular meal, prepared more meals to diversity their household diets, or wanted to improve the diets of their children.

**Table 5:** Reasons for and against cooking extra food and storing it for later use (N=127)

Theme	Examples of caregivers' reasons for cooking extra food for later use (n = 108)
Social norm (50%)	*We have always prepared food this way, ever since I was a child. It is a traditional practice that has been passed on from generation to generation*. *We do not know any other way of preparing* [food], *this is how we were taught by our parents and grandparents*. *Whenever our children get hungry, we normally do not cook more food, but there must always be some food there for them to eat anytime. Even when we were young, there was always food kept which we would eat anytime*. *It is* [our] *culture to ensure you have some food ready just in case a visitor came by*.
Save fuel (39.8%)	*Our resources are limited and are not enough for buying fuel for cooking every time*. *Firewood and charcoal are expensive*
Avoid wastage (10.2%)	*When food is cooked in excess, it is not thrown away. It is kept for later use to avoid food wastage*. *We do not throw away food, we keep it so that the children can eat it later when they are hungry instead of wasting it*.
Laziness (0.9%)	*I just don't want to cook many times in a day, it is easy if I cook only once and rest*. *I feel lazy to prepare meals several times a day*.
**Theme**	**Examples of caregivers' reasons against storing food for later use (n =19)**
Cook Just enough (70.5%)	*The food cooked is all eaten as it is usually adequate for the large family sizes*. *I cook only what is enough for a meal to avoid having to throw the remainder away*.
Diversity of meals (11.8)	*So that I will be able to change the menus for the next meals in the day*. *To avoid monotonous meals*.
Monitor child's diet (17.7%)	*I am trying to control the children's high appetite and eating habits*. *The baby has just started on complementary feeding and eats very little thus no point in cooking a lot of food*.

Overall, the major food safety hazards in food preparation were associated with the storage and reheating (warming) of the ‘leftover food.’ Table 6 summarises risky practices in preparation of the foods that were commonly used as complementary feeds for children 6-23 months of age. Observations and caregivers' self-reports also indicate that the leftover food was left in the saucepan or put on plates for later use. However, the ‘leftover’ food was not warmed before serving it to children and thus posing a safety concern. In most households, the leftover main staples (mostly maize meal loaf or boiled sweet potatoes and cassava) were served cold and only the sauces or soups were warmed before feeding the child. Some caregivers reported adding cold untreated water to cooked food when reheating it; yet the majority (85%) reported that food was often not heated long enough.

**Table 6:** Common complementary food preparation methods and threats to food safety

Form food eaten	Common preparation method	Threats to food safety
Maize porridge (gruel)	Refined, white maize flour was mixed in water to make a paste which was then boiled to make a gruel (porridge). Gruel was kept in flasks or cups and used throughout the day.	Porridge kept in cups was not warmed before serving the children. Some flasks were not easy to wash. Some flasks did not keep the porridge hot enough deter microbial spoilage.
*Posho* (maize bread or stiff porridge)	Refined, white maize flour was mingled in boiling water to make bread. The hot bread was commonly complemented with sauce or soup made from pulses, vegetables, and meats.	Inappropriate storage Majority of the caregivers did not warm the leftovers before serving their children.
Boiled fresh maize on the cob	Mature, fresh maize on the cob was boiled or steamed in its husks, then served hot or cold.	Usually purchased ready to eat along dusty streets. Sometimes unsold maize was kept for more than day.
Roasted fresh maize on the cob	Mature, fresh maize on the cob was removed from the husks and roasted directly on charcoal in fireplace or over a charcoal stove till all seeds are golden brown. This was commonly used as snack.	Usually purchased ready to eat along dusty streets.
*Spaghetti and Rice*	*Spaghetti* (commonly referred to as *Macaroni*) was a treasured food among young children. They cut it into small pieces of about 5cm long and placed in boiling water. A little salt was added to taste. The pasta was boiled till soft, then served. Rice was also boiled in a comparable way; some households washed the rice before cooking while others did not. Alternatively, cooking oil was added to a preheated pan, then onions are added to the hot oil and cooked till brown before chopped tomatoes, oil, spices, and salt were added. Then water was added and allowed to boil before cut pieces of the spaghetti, or the rice was added and left to boil till soft to the touch.	Inappropriate storage The commonest risky practice observed was when the left-over food was kept either for later or because it was not finished, children were seen often going to pick pieces from the plates or cooking pots (where the food was kept) and eating; without washing their hands. Each time they got hungry during their play time, they would just go and pick the food leftover to snack on.
Sweet potatoes and cassava	*Sweet potatoes and cassava* are boiled or steamed as part of household meal and sometimes roasted over charcoal or in the hot ash of burning *firewood*.	The caregivers did not warm these leftovers before serving their children.
Commonly purchased snack foods	Snacks such as doughnuts, *mandazi, chapatti*, cakes, bread rolls (buns) and tea biscuits were purchased as ready-to-eat snacks	These snacks were prepared and sold in unsanitary environments such as by the roadside At the selling point, the snacks were not protected from the environment or by peddlers in uncovered baskets After buying, these snacks (apart from tea biscuits) were wrapped in old used newspapers, scrap paper, or scrap paper bags
**Form of food eaten**	**Common preparation method**	**Undesirable Handling practices (after cooking)**
**Vegetables**	Leafy vegetables such as *boo, malakwang* were sorted on a straw winnower before cooking. Other vegetables such as cabbage, and egg plants were cut into small pieces and were then be washed (sometimes used unwashed) and then fried, boiled or added to groundnut or *simsim* (sesame) paste to make sauce	The cow dung commonly used to fill gaps in the winnowers predisposes the vegetables to faecal contamination Washing vegetables after cutting them known to foster leaching of nutrients while using unwashed vegetables could contaminate food The leftovers were stored in either partially covered or open saucepans or plates to reduce quick spoilage. Inadequate warming (the food/sauce/stew was not allowed to boil). It was warmed slightly so that it was comfortable for children to consume right after warming.
	Onions and tomatoes are fried with some oil or margarine and water plus salt are added to make sauce.	
Legumes, nuts, and seeds	Grains were sorted on local trays/winnowers which were made by weaving dried papyrus reeds that have and finished with cow dung to fill up the spaces and smoothen the surface. Fresh or dried beans and peas were cooked without prior soaking or any treatments. Cooked beans or peas were then used to make stews or pottages and sauces or soups. Legumes were commonly fried with tomatoes and onions or mixed in groundnut or *simsim* to make sauces or soups that were used to complement the staple foods, notably *posho*, cassava, sweet potatoes, millet, and rice.	The practice of using local trays (winnowers) increased the risk of contamination with helminthic ova: especially when these trays were used to handle foods such as flour or leafy vegetables that do not require cooking for a long time; to handle foods that were eaten raw (notably fruit), or to cover cooked food. Inappropriate storage which allowed dust and microbes from the air to get into the food. The leftovers were stored in either partially covered or open saucepans or plates. Inadequate warming (the food/sauce/stew was not allowed to boil before it was served to the children). The inadequately reheated sauce was mixed with cold leftover staple food and given to the child to eat. The storage and alternate uses of the frond used to stir *simsim* during roasting was likely to introduce helminthic eggs to the roasted *simsim* especially when used to stir nuts or *simsim* as it cooled down.
Groundnuts (Peanut) and *Simsim* (Sesame)	Raw groundnut powder was mixed in water and cooked for over an hour before serving. Groundnuts & *simsim* were also roasted in pans using either a wooden spoon or a broom-like palm frond and then *g*round into a powder or paste used to make a sauce or to thicken other sauces. The paste was mixed in water and cooked for about 5 minutes. Paste which is made by grinding roasted nuts or *simsim* can be eaten without cooking as spread on bread, leftover cassava, and banana fruits; or is added to foods such as beans, peas, fish, meat, chicken, vegetables, bananas (*matooke*), cassava, and boiled potatoes during the last five minutes before the food is taken off the fire. Groundnut paste was used to thicken sauces, boiled in water, or added to other foods and vegetables	
Dairy products	Milk was mixed with some water and boiled in a pan. Tea leaves and/or sugar were sometimes added.	Boiled milk or milk tea was kept in a flask or cup for later use. It was not warmed at the time of serving the child.

Additionally, the practice of making cooked food easily accessible to older children to eat whenever they are hungry or feel like eating improves the number of eating occasions (meal frequency) but may pose a food safety hazard. Notably, some older children were observed coming from playing and dashing into the house to retrieve the stored food and eat it right away without washing their hands.

## Discussion

Generally, children's feeding practices were adequate to support normal growth since most children were breastfeeding; however, the rates of exclusive breastfeeding among children 0-6 months and continued breastfeeding among children 12-23 months remain low as has been observed in similar contexts^17^–^20^. The rate of continued breastfeeding (70%) among children 6-23 months was higher than the national average^21^,^22^, hence is commendable, but needs more improvement.

Assessment of children's diets using the child feeding index (CFI) indicate that most children met the minimum requirements for breastfeeding and complementary feeding; however, the observed patterns may not assure optimal nutritional status to support normal growth especially in contexts where children are at high risk of infections. Inappropriate child-feeding practices that are likely to compromise nutritional status include: not adhering to exclusive breastfeeding during the child's first 6 months of life, inadequate number of meals per day, inadequate dietary diversity and infrequent inclusion of animal source foods in children's diets. The different reasons that have been cited for these inappropriate feeding practices in Uganda include lack of knowledge of the appropriate feeding practices, heavy workloads for the mothers/caregivers, and the influence of culture^1^–^4^; however, nutrition education has potential to address these factors^23^–^26^. This study also highlights access to cooking fuel (firewood and charcoal) as one of the major factors that limits the number of meals prepared at a household level.

As has been reported among other populations in northern Uganda^25^,^27^,^28^, children are mostly complemented with the plant-based foods that households produce and consume, which limits dietary diversification. These practices have also been reported in other parts of the country^26^,^29^,^30^, and this is a major concern since plant-based diets low in animal-source foods, fruits and vegetables are likely to be insufficient in critical micronutrients such as zinc, iron, and calcium which are needed in high amounts by rapidly growing young children^31^. There are diverse factors that explain the limited consumption of animal-source foods such as meat, eggs, fish, and poultry^29^–^32^; however, the cost of these foods seems to have been the major factor limiting their consumption in this semi-urban, limited resource context.

Besides dietary patterns, inappropriate food preparation and handling practices were identified as threats to child nutrition. The major risky food preparation and handling practices identified at household level included: age-inappropriate consistency of complementary foods, unsanitary storage of food cooked for later use, and addition of untreated and unboiled water to cooked food. Other potentially risky handling practices were also noted in food acquisition whereby ready-to-eat vended snacks were not properly handled, were sold in unsafe packaging materials, or were vended in unsanitary environments. Studies have shown that these practices not only result in microbial contamination of the food but also influence the incidence and prevalence of diarrhoea among young children 8–10, which increases children's risk of under-nutrition.

The long storage of cooked food (especially overnight) is also a major concern since studies have shown that the microbial load of the cooked food increases with storage time^8^–^10^; and when reheated, the microbial load reduces but not to safe levels due to presence of thermo-tolerant coliform bacteria. This is a major health concern since caregivers reported that food was never heated to temperatures high enough to reduce the bacterial load. Conversely, caregivers included in this study were more likely to introduce these thermo-tolerant coliforms into children's foods by not washing their own hands after helping a child that has defecated or soiled themselves, and not washing children's hands before eating^32^. These practices are likely to increase the risk of diarrheal infections and the associated malnutrition since common thermo-tolerant coliforms belong to the faecal coliform group - which include diarrhoea-causing bacteria such as *E. coli*^33^.

This study was limited by lack of data on the determinants of appropriate complementary feeding and food handling practices, especially since diet quality and food handling practices were associated with malnutrition^32^. Since the caregivers in this study had better adherence to the WHO IYCF guidelines than reported by other researchers^17^,^34^, and since a higher proportion of children had better diets (indicated by breastfeeding patterns, MDD, MMF, and total CFI) than has been reported nationally^21^,^22^,^35^ and in Northern region^27^,^28^, there is need for more research to determine the facilitators of adequate child-feeding and barriers to appropriate food handling practices in this study population.

## Conclusion

This study indicates that young children (6-23 months of age) in Adyel division in Lira district were provided nutritionally acceptable diets; however, food preparation and handling practices are likely to compromise children's nutritional status. Dietary diversity was limited by the low frequency of meal preparation resulting from the reliance on leftover food. Hence, improving child-feeding practices in this study area requires improving access to complementary feeds that require minimum preparation time and access to safe ready-to-eat nutrient-dense snacks. The food-handling and child-feeding practices reported by caregivers also highlights the need for nutrition education and skills training to improve the caregivers' complementary feeding and food handling practices within their socio-economic and cultural context.
